# The combination of R2R3-MYB gene *AmRosea1* and hairy root culture is a useful tool for rapidly induction and production of anthocyanins in *Antirrhinum majus* L

**DOI:** 10.1186/s13568-021-01286-6

**Published:** 2021-09-14

**Authors:** Chunlan Piao, Jinguo Wu, Min-Long Cui

**Affiliations:** 1 College of Horticulture Science, Zhejiang A & F University, Linan, 311300 China; 2 Institute of Virology and Biotechnology, Zhejiang Academy of Agricultural Sciences, Hangzhou, 310021 China

**Keywords:** *Antirrhinum majus* L., R2R3-MYB gene *AmRosea1*, Transformed hairy roots, Activation of anthocyanin biosynthesis, Anthocyanin content

## Abstract

Anthocyanins are the largest group of water-soluble pigments and beneficial for human health. Although most plants roots have the potential to express natural biosynthesis pathways required to produce specialized metabolites such as anthocyanins, the anthocyanin synthesis is specifically silenced in roots. To explore the molecular mechanism of absence and production ability of anthocyanin in the roots, investigated the effect of a bHLH gene *AmDelila*, and an R2R3-MYB gene *AmRosea1*, which are the master regulators of anthocyanin biosynthesis in *Antirrhinum majus* flowers, by expressing these genes in transformed hairy roots of *A. majus*. Co-ectopic expression of both *AmDelila* and *AmRosea1* significantly upregulated the expression of the key target structural genes in the anthocyanin biosynthesis pathway. Furthermore, this resulted in strongly enhanced anthocyanin accumulation in transformed hairy roots. Ectopic expression of *AmDelila* alone did not gives rise to any significant anthocyanin accumulation, however, ectopic expression of *AmRosea1* alone clearly upregulated expression of the main structural genes as well as greatly promoted anthocyanin accumulation in transformed hairy roots, where the contents reached 0.773–2.064 mg/g fresh weight. These results suggest that *AmRosea1* plays a key role in the regulatory network in controlling the initiation of anthocyanin biosynthesis in roots, and the combination of *AmRosea1* and hairy root culture is a powerful tool to study and production of anthocyanins in the roots of *A. majus*.

**Supplementary Information:**

The online version contains supplementary material available at 10.1186/s13568-021-01286-6.

## Introduction

The plants including *Antirrhinum majus* are capable of biosynthesizing a wide variety of secondary metabolites including flavonoids, polysaccharides, fatty acids, vitamins, alkaloids, terpenoids and iridoid glycosides (Mehrotra et al. 2015; Jeziorek et al. 2018; Yousefian et al. 2020; Seo et al. 2020; Roy 2021). Among them, anthocyanins are flavonoids that a class of useful secondary metabolites play inhibit cancer cell proliferation and to serve as antioxidants promote human health (Tavsan and Kayali 2019; Kopustinskiene et al. 2020). The anthocyanins are found in the leaves, stems, flowers and fruits, however absence in the root of most plants. Moreover, despite the anthocyanin biosynthesis pathway in flowers, leaves and fruits of plants is well understood (Martin et al. 1991; Zhang et al. 2014), the molecular basis for the absence of anthocyanin accumulation in the roots remains unclear.

The plants are natural producers of many important pharmacologically active secondary components including anthocyanins (Sharma et al. 2018). However, plant propagation and tissue culture to overproduction of some useful pharma molecules has limitations, especially where the synthesis of these molecules is affected by their complicated developmental regulation in the different cells, tissues, organs and ages in plants. Biotechnological approaches involving *Agrobacterium*-transformed tissue culture have the potential to overcome this. *Agrobacterium rhizogenes* has the ability to transfer its T-DNA from the root-inducing (Ri) plasmid to the host plant genome, thereby inducing the formation of hairy roots. The transformed hairy roots are rapidly and efficiently induced from explant tissues of the host plants and the culture procedure is very simple and can be maintained for a long period; the hairy roots show rapid growth rates and similar genetic characteristics to those of normal roots. Moreover, the hairy roots appear to produce the same spectrum of metabolites as do roots in planta, in addition to synthesizing novel compounds (Ritala et al. 2014; Mehrotra et al. 2015; Thakore and Srivastava 2017; Roy 2021). Therefore, hairy root culture represents a useful tool for studying molecular mechanisms of secondary metabolism, the molecular function of the genes involved and provide a reliable platform for production specific components by bioengineering (Gao et al. 2013; Ghorbani 2017).

Anthocyanins are flavonoid pigments. The biosynthetic pathway of anthocyanin is well studied and the main structural genes involved in this pathway such as chalcone synthase (*CHS*), chalcone isomerase (*CHI*), flavanone 3-hydroxylase (*F3H*), flavonoid 3′-hydroxylase (*F3′H*), dihydroflavonol 4-reductase (*DFR*), and anthocyanidin synthase (*ANS*) have been isolated and characterized in *A. majus, Arabidopsis* and petunia (Martin et al. 1991; Kitamura et al. 2004; Ai et al. 2016). The structural genes are regulated by transcription factors, including R2R3-MYB, basic helix-loop-helix (bHLH), and WD40 proteins (Ramsay and Glover 2005; Gonzalez et al. 2008; Albert et al. 2014). Among these, the R2R3-MYB genes play significant roles in regulate of anthocyanin biosynthesis process (Gao et al. 2013; Borevitz et al. 2000; Naing and Kim 2018).

*Antirrhinum majus* is a medicinal plant as well as has been used particularly as a model system for the molecular analysis of floral pigmentation (Martin et al. 1991; Saqallah et al. 2018; Seo et al. 2020). In *A. majus*, the structural genes encoding the enzymes of anthocyanin biosynthetic pathway have been well characterized and identified genetically, namely, *AmCHS*, *AmF3H*, *AmF3′H*, *AmDFR*, and *AmAS* (Additional file 1: Fig. S1) (Martin et al. 1985, 1991; Sommer and Saedler 1986). These genes are divided into two groups, early biosynthetic genes (EBGs), including *AmCHS*, *AmCHI*, *AmF3H*, and *AmF3'H*; and late biosynthetic genes (LBGs), including *AmDFR*, *AmANS*, and *AmUFCT* (Pelletier et al. 1997; Winkel-Shirley 2001). In *Antirrhinum*, each of the two groups has been found to be mainly co-regulated by different regulators, including an bHLH transcription factor, *AmDelila* (*AmDEL*), and three R2R3-MYB transcription factors, namely, *AmRosea1(AmROS1)*, *AmRosea2* (*AmROS2*), and *AmVenosa* (*AmVE*) (Almeida et al. 1989; Schwinn et al. 2006; Shang et al. 2011). In flowers of *A. majus*, *AmDelila* affects pigmentation in the corolla tube; *AmRosea1* affects the pattern and intensity of pigmentation in the lobes and tubes; and *AmVenosa* affects pigmentation of the epidermis overlying the veins in the lobes and tubes. In addition, transcription factors are required for the activation of expression of the late biosynthetic genes, including *AmDFR*, *AmAS* and *AmUFGT*, in the corolla tube (Goodrich et al. 1992; Martin and Gerats 1993).

In the present study, we investigated the molecular mechanism of absence and production ability of anthocyanins in the transformed hairy roots of *A. majus*, and discuss the usefulness of combination of the R2R3-MYB gene *AmRosea1* and hairy root culture method is a powerful tool to control of the secondary metabolic pathway and product of anthocyanins in the root of *A. majus.*

## Materials and methods

### Plant material and growth conditions

The seeds of *Antirrhinum* JI 7 were used in this study (provided by Lucy Copsey and Professor Enrico Coen, John Innes Centre, UK). The seeds were surface-sterilized by brief rinsing in 70% (v/v) ethanol, followed by sterilized in a 2% (v/v) solution of sodium hypochlorite for 10 min and washed five times using sterilized water. The sterilized seeds were germinated on solid MS medium (Murashige and Skoog 1962) in a growth room at 25 °C, 16 h light/8 h dark photoperiod. Four-week-old seedlings were used for transformation.

### *Agrobacterium* strains and plasmids

**Fig. 1:**
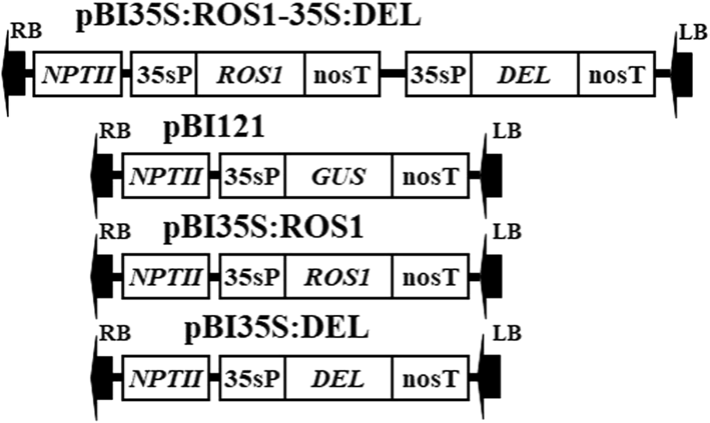
T-DNA regions of the binary vectors used in this study. nosT, terminator of the nopaline synthase gene; *NPT II*, neomycin phosphotransferase gene; 35sP, cauliflower mosaic virus 35 s promoter; *ROS1*, R2R3-MYB gene *AmRosea1* of *A. majus*; *DEL*, bHLH gene *AmDelila* of *A. majus*; *GUS*, β-glucuronidase gene; RB, right border of T-DNA; LB, left border of T-DNA

The plant expression binary vectors of pBI35S:ROS1-35S:DEL, pBI35S:ROS1, pBI35S:DEL, and pBI121 (Fig. 1) were introduced into *Agrobacterium rhizogenes* strain of AR1193 (Weidi Biotech Co. Ltd. Shanghai, China) by electroporation (Shen and Forde 1989), and used in this study.

### *A. rhizogenes*-mediated transformation of *A. majus*

The transformation of *A. majus* was performed according to the methods of Senior et al. (1995) and Cui et al. (2001). The *A. rhizogenes* strains were grown in 5 mL of liquid LB medium containing 50 mg/L kanamycin and 100 mg/L rifampicin at 28 °C, shaking at 200 rpm for 24 h. The *Agrobacterium* cultures were diluted 40-fold with liquid MS medium before inoculation. About 1 cm long hypocotyl segments of *A. majus* were inoculated with the diluted *Agrobacterium* suspensions for 8–10 min, and transferred to a solid co-cultivation MS medium containing 1 mg/L zeatin, 0.1 mg/L NAA (1-Naphthaleneacetic acid), and 20 μM acetosyringone. About 50 hypocotyl segments for each treatment were used. After 3 days of co-cultivation, the infected hypocotyl segments were transferred to solid MS medium containing 50 mg/L kanamycin and 250 mg/L cefotaxime and induced transformed hairy roots. The obtained adventitious roots were transferred to fresh solid MS medium containing 250 mg/L of cefotaxime and selected transformed hairy roots were seen by visible coloration and harvested for PCR analysis. The selected hairy roots were maintained at 25 °C under a 16 h light/8 h dark photoperiod condition.

### Polymerase chain reaction analysis

Genomic DNA was extracted from a non-transformed root, transformed hairy roots of pBI121, pBI35S:ROS1-35S:DEL, pBI35S:ROS1 and pBI35S:DEL, respectively, according to the CTAB (Hexadecyl trimethyl ammonium Bromide) method (Rogers and Bendich 1985). The primer sets of *NptII*, *AmROS1*, and *AmDEL* were used for amplification (Table 1). PCR analysis was performed by using an ABI 2720 PCR machine, with 20 μL of reaction mixtures containing 50 ng genome DNA, 10 pmol of each primer, and 1 unit of *Taq* polymerase (Takara, Dalian, Japan). The following PCR conditions were used: an initial denaturation step at 94 °C for 3 min; followed by 35 cycles each at 94 °C for 1 min, 58–62 °C for 1 min, and 72 °C for 2 min; and a final extension step at 72 °C for 10 min. Amplified DNA bands were analyzed by using 1.0% (w/v) agarose gel electrophoresis at 100 V for 30 min, followed by staining with ethidium bromide and observation under UV illumination.

**Table 1:** Primers used for detection of transformed hairy roots and RT-PCR analysis in this study

Genes	Forward primers	Reverse primers
AmCHS	GCAGCAGCGGTTATAGTTG	CGCCGAAGACTTCCTCATT
AmF3H	TGACTGATATGGCACGAGAGT	TGATCCTGGAGCAGCAAAGTA
AmDFR	GTGCGATTGACACTTGCC	CTGCCATCAGTATGATCGTTTG
AmANS	GCATTTGATTAACCACGGTGT	CAATAACAACACCACCACCAT
AmUBI	ATTGGTGCTGAGGTTGAGA	ACAACTGACTCCAGCAAACG
AmROS1	ATGGAAAAGAATTGTCGTGG	TTAATTTCCAATTTGTTGGG
AmDEL	ATGGCTACTGGTATCCAAAA	GAGTGCTGTGCATACAATTA
NPT II	AGATGGATTGCACGCAGGTTC	GTGGTCGAATGGGCAGGTAG

### Expression analysis of the genes involved in the anthocyanin biosynthetic pathway in hairy roots

Total RNA was extracted from 0.1g samples of a non-transformed root and the transformed hairy roots with pBI21, pBI35S:ROS1-35S:DEL, pBI35S:ROS1 and pBI35S:DEL, respectively*,* use the SV Total RNA Isolation System and RNase-free DNase (Promega, Beijing, USA). First-strand cDNA was synthesized from 2 µg of total RNA using a Superscript III First Strand cDNA Synthesis Kit (Invitrogen, Shanghai, USA). Semi-quantitative RT-PCR analyses were carried out using *AmROS1*, *AmDEL*, *AmCHS*, *AmF3H*, *AmDFR*, *and AmANS* gene-specific primers (Table 1) and the *ubiquitin* genes of *A. majus* (AmUBI) as positive control. The PCR conditions are: a preliminary denaturation step at 95 °C for 5 min; followed by 30 cycles of denaturation at 94 °C for 30 s, annealing at 58–62 °C for 1 min and extension at 72 °C for 1 min; and a final extension step at 72 °C for 10 min. The RT-PCR experiments were repeated at least two times independently, and the PCR products were confirmed by sequencing.

### Quantification of anthocyanins

A hairy root transformed with pBI121 as a control and five independent hairy roots transformed with pBI35S:ROS1 were ground to a fine powder in liquid nitrogen, and 100 mg of the powder was extracted with 1 mL acidic methanol (1% hydrochloric acid, w/v) at room temperature for 12 h with moderate shaking. After centrifugation at 12,000 rpm for 10 min, 800 mL of the supernatant was added to 4 mL of acidic methanol. The absorbance at 530 and 657 nm was determined using a spectrophotometer (UV757CRT, Shanghai precision and scientific instrument Co. Ltd., China), and the relative level of anthocyanin was calculated using the equation ODA530–(0.25 X ODA657) (Rabino and Mancinelli 1986). Each sample was tested three times. Error bars indicate the standard deviation (SD) values of the average anthocyanin contents.

## Results

### Expression analysis of *AmDelila* and *AmRosea1 *in* A. majus*

**Fig. 2:**
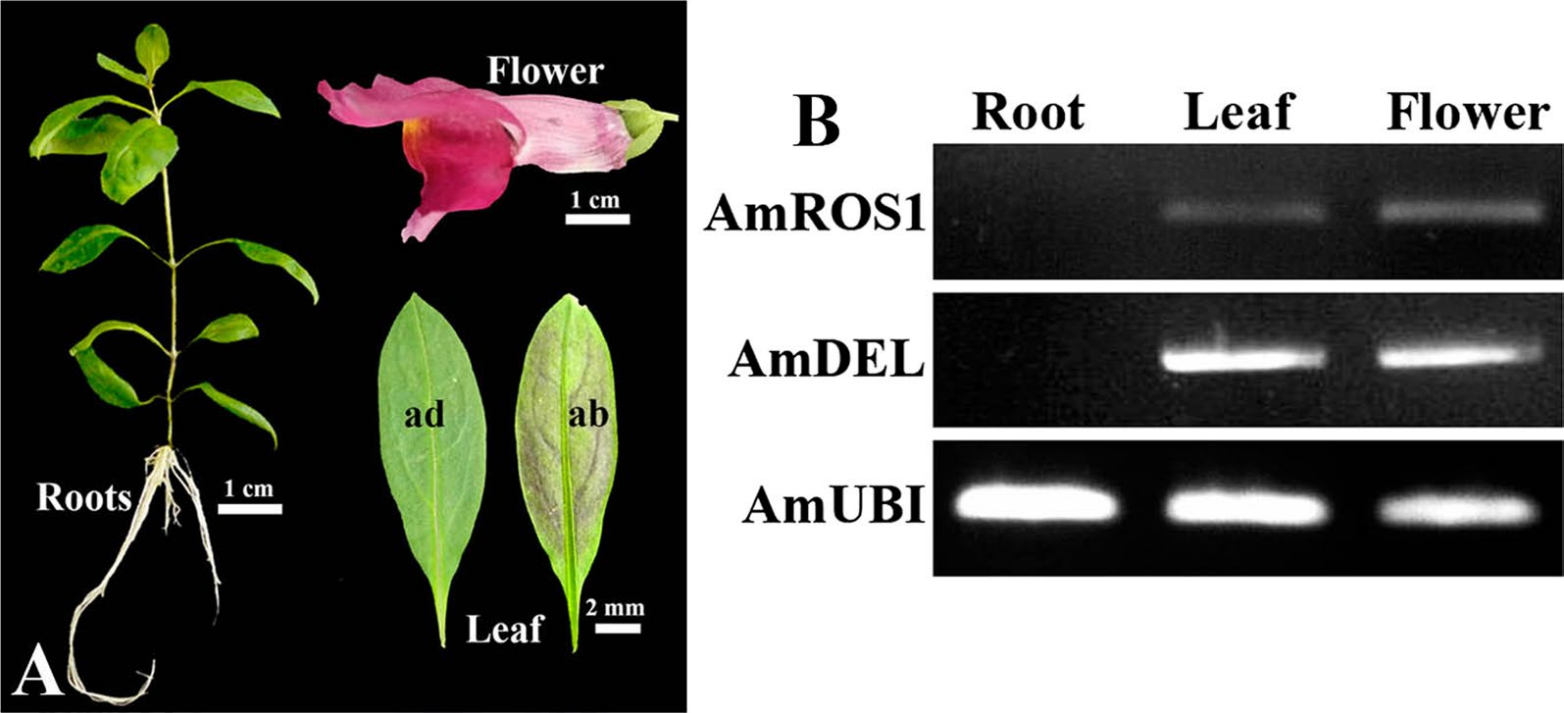
Comparison of colorations in the roots, leaves, and flowers of *A. majus* JI 7 and RT-PCR analysis of *AmRosea1* and *AmDelila* expression. **A** Six-week-old seedling, mature leaves and flower of *A. majus*; **B** Semi-quantitative RT-PCR-based expression analysis of *AmRosea1* (*AmROS1*) and *AmDelila* (*AmDEL*) in the roots, leaves, and flowers. Scale bar = 1 cm. ad, adaxial of leaf; ab, abaxial of leaf

Despite the leaves of *A. majus* exhibited red coloration in the abaxial parts when grown under light conditions, the roots did not display any color accumulation, regardless of light conditions (Fig. 2A). To determine the molecular mechanism responsible for the absence of anthocyanin accumulation in the roots, investigated the expression of the *AmDelila* and *AmRosea1 *in the roots, red-colored leaves, and flowers of *A. majus* JI 7 (Fig. 2A), by using semi-quantitative RT-PCR analysis (Fig. 2B). The visible expression of both *AmDelila* and *AmRosea1* were detected in the flowers and leaves, however, the *AmDelila* and *AmRosea1* expression were not observed in the roots (Fig. 2B). Therefore, absence of anthocyanin pigmentation in the roots of *A. majus* may be affected by silence of *AmDelila*- or *AmRosea1*-like regulatory genes.

### Simultaneous expression of *AmRosea1* and *AmDelila* promotes anthocyanin accumulation in transformed hairy roots of *A. majus*

**Fig. 3:**
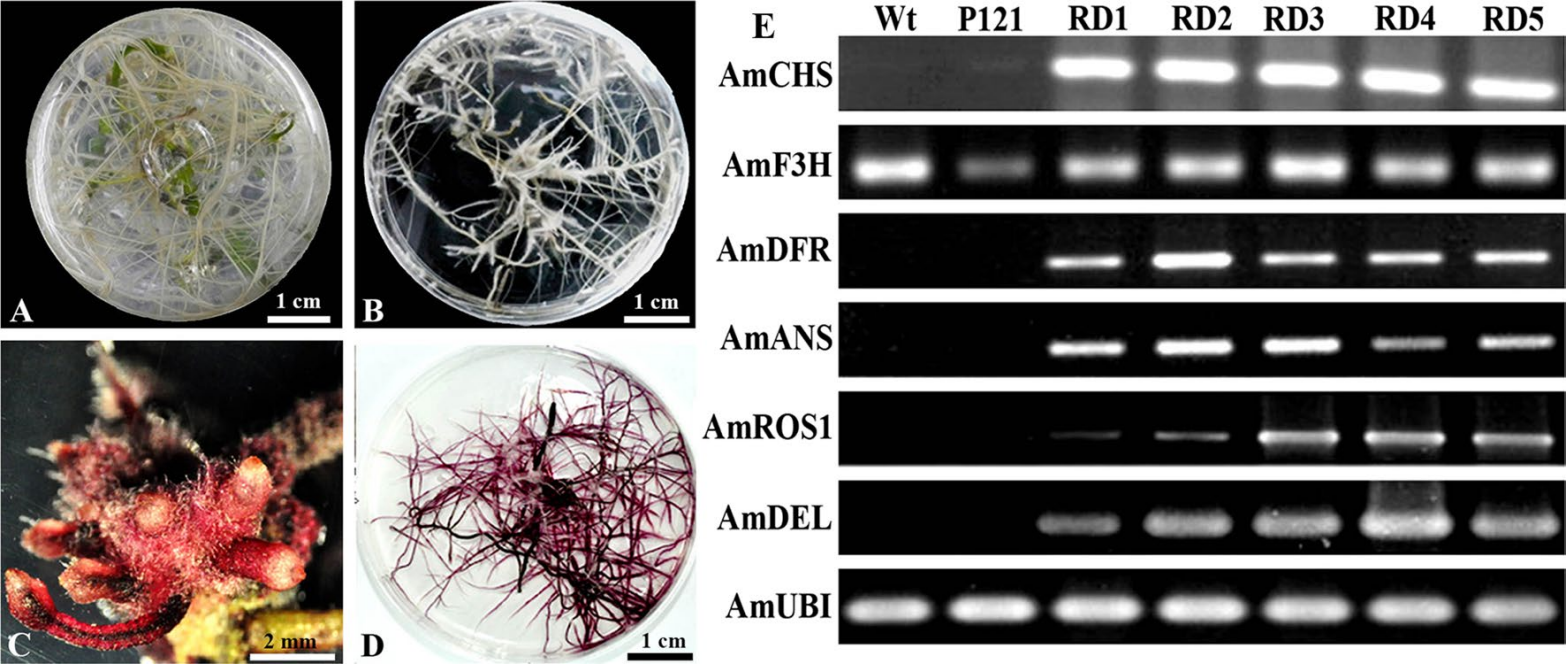
Comparison of coloration and expression analysis of anthocyanin-related genes by PCR in normal roots (wild type transformed hairy roots with AR1193/pBI121), and transformed hairy roots with AR1193/pBI35S:ROS1-35S:DEL. **A** Normal root of 6-week-old seedlings on MS medium; **B** A negative control hairy root transformed with AR1193/pBI121 (P121) after 6 weeks of culture on MS + 200 mg·L^−1^ of cefotaxime; **C** Transformed hairy roots emerging from the hypocotyl segment after 3 weeks infection with AR1193/pBI35S:ROS1-35S:DEL; **D** Transformed hairy roots of with AR1193/pBI35S:ROS1-35S:DEL after 6 weeks culture on MS + 200 mg·L^−1^ of cefotaxime. **E** Analysis of *AmCHS*, *AmF3H*, *AmDFR* and *AmANS* expression levels from a transformed hairy root with AR1193/pBI121 and five transformed hairy root lines with AR1193/pBI35S:ROS1-35S:DEL by semi-quantitative RT-PCR. P121, A negative control hairy root transformed with AR1193/pBI121; RD1 to 5, Five transformed hairy root lines with AR1193/pBI35S:ROS1-35S:DEL

To test hypothesis, we used the *A. rhizogenes* strains AR1193/pBI35S:ROS1-35S:DEL with harboring both *Delila* and *Rosea1* genes under the control of the 35S promoter, respectively, and AR1193/pBI121 (35S:*GUS*) as a negative control to transform *A. majus* JI 7. After 3 weeks of infection, transformed non-pigmented hairy roots were emerged from the wounded end of hypocotyl segments co-cultivated with AR1193/pBI121 (data not shown). Despite of these hairy roots were similar to non-transformed root showed no any color accumulation, these roots revealed rapid elongation when maintained under 16 h light/8 h dark conditions (Fig. 3A, B)*.* In contrast, highly pigmented hairy roots emerged from hypocotyl segments transformed with AR1193/pBI35S:ROS1-35S:DEL (Fig. 3C, D). These roots appeared later than non-pigmented hairy roots (Fig. 3C), and the elongation was slower than for non-pigmented hairy roots when maintained under 16 h light/8 h dark condition (Fig. 3D).

To analyse the mechanism for the *AmDelila*/*AmRosea1* effect, we looked at the expression of target structural genes of the anthocyanin biosynthetic pathway. We investigated the expression patterning of the main structural genes, *AmCHS*, *AmF3H*, *AmDFR*, and *AmANS*, from hairy roots transformed with pBI35S:ROS1-35S:DEL and a negative hairy root transformed with AR1193/pBI121 (P121), by using semi-quantitative RT-PCR analysis (Fig. 3E). Except of *AmF3H*, the expressions of *AmCHS*, *AmDFR* and *AmANS* were dramatically upregulated in the hairy roots transformed with pBI35S:ROS1-35S:DEL, and that the expression pattern correlated with the anthocyanin accumulations in the hairy roots (Fig. 3D, E). In contrast we did not see any significant expression of *AmDFR* and *AmANS* in negative control hairy roots of P121 (Fig. 3B). Our results indicate that the combination of *AmDelila* and *AmRosea1* are able to activate the expression of the structural genes involved in the anthocyanin biosynthetic pathway to enhanced anthocyanin accumulation in the hairy roots of *A. majus*.

### Ectopic expression of *AmRosea1* alone activates anthocyanin synthesis in transformed hairy roots

**Fig. 4:**
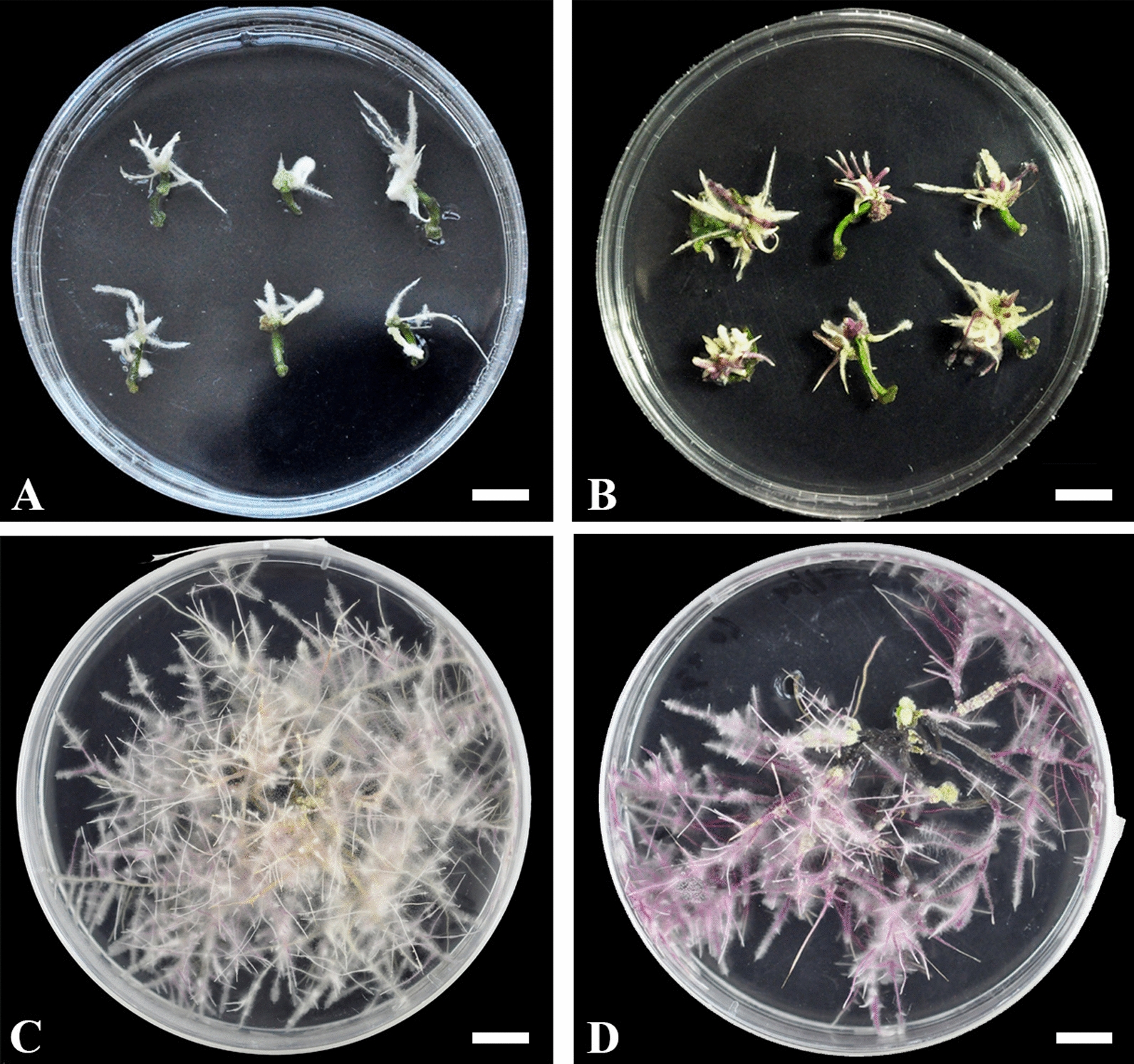
Phenotypic comparison of coloration in hairy roots transformed with AR1193/pBI35S:DEL and AR1193/pBI35S:ROS1. **A** Transformed hairy roots emerging from the hypocotyl segments after 3 weeks infection with AR1193/pBI35S:DEL; **B** Transformed hairy roots emerging from the hypocotyl segments after 3 weeks infection with AR1193/pBI35S:ROS1; **C** Pale-red colored hairy roots of transformed with AR1193/ pBI35S:ROS1 after 6 weeks of culture on MS + 200 mg·L^−1^ cefotaxime; **D** Deep-red colored hairy roots of transformed with AR1193/pBI35S:ROS1 after 6 weeks of culture on MS + 200 mg·L^−1^ cefotaxime. Scale bar = 1 cm

To investigate the contribution of each gene, AR1193/pBI35S:DEL, and the negative control AR1193/pBI121 were transformed into 3-week-old hypocotyls of *A. majus* JI 7, respectively. After 3 weeks of infection, a lot of independent hairy roots emerged from hypocotyl segments transformed with pBI35S:DEL (Fig. 4A). These hairy roots were maintained for 6 weeks under 16 h light/8 h dark condition, but remained similarly non-pigmented to the hairy roots of AR1193/pBI121 (P121). These results indicate that *AmDelila* alone does not appear to have the ability to stimulate anthocyanin synthesis in roots of *A. majus*.

**Fig. 5:**
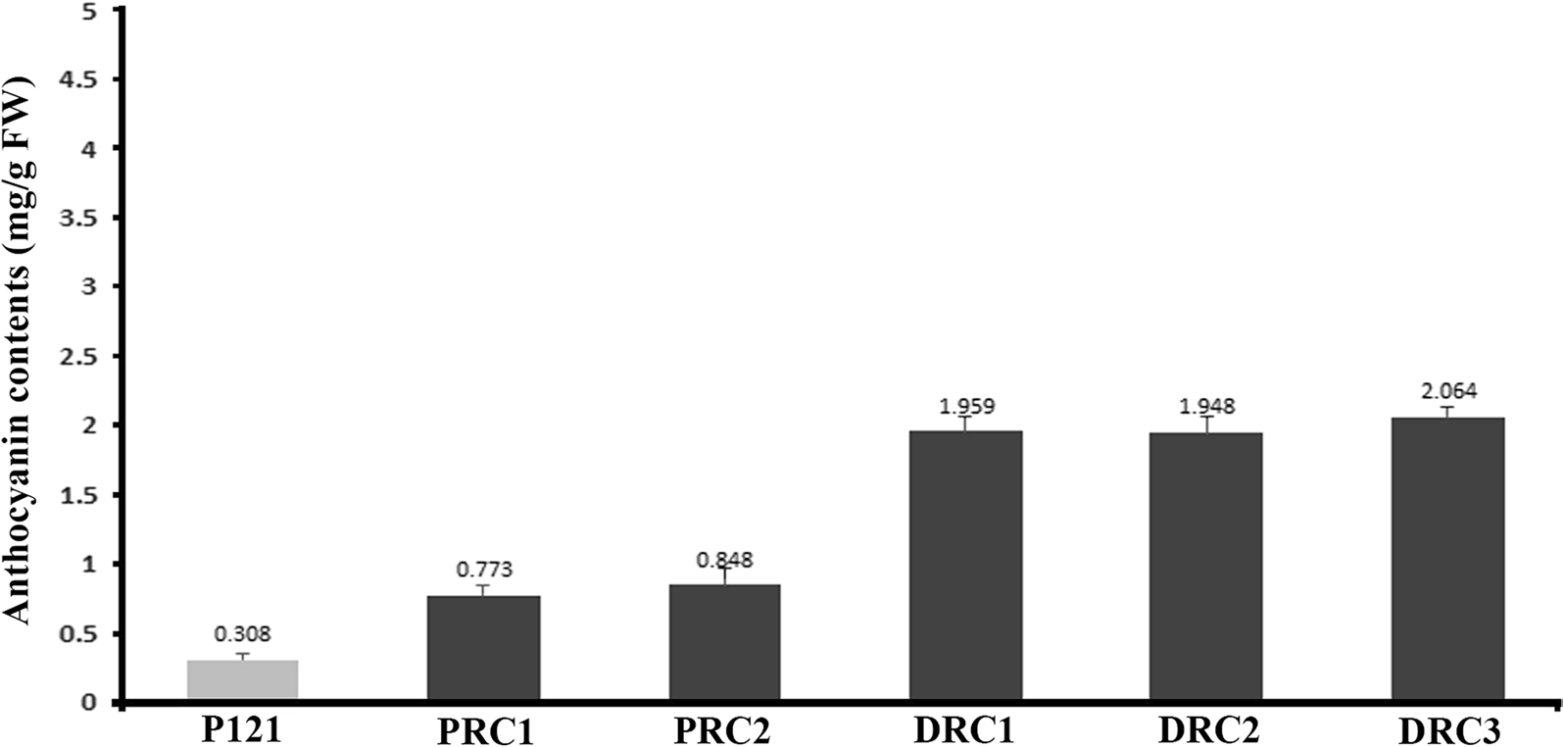
Analysis of total anthocyanin concentrations from transformed hairy roots with AR1193/pBI35S:ROS1 and a negative control hairy root transformed with AR1193/pBI121. Total anthocyanin was extracted from two pale colored hairy roots PRC1 (Fig. 4C) and PRC2 (Additional file 2: Fig. S2A), three deep colored hairy roots DRC1 (Additional file 2: Fig. S2B), DRC2 (Additional file 2: Fig. S2C) and DRC3 (Fig. 4D) and a control hairy root P121 (Fig. 2B), respectively. The anthocyanin content was measured using a UV spectrometer. Means of three replicates with error bars indicating standard error (± SD)

Next, investigate the ability of *AmRosea1,* the AR1193/pBI35S:ROS1 transformed into 3-week-old hypocotyls of *A. majus* JI 7. After 3 weeks of infection, a lot of independent hairy roots emerged from hypocotyl segments transformed with pBI35S:ROS1 and many hairy roots showed pale or purple red pigmentation (Fig. 4B). Among these, some pale-red colored hairy roots (PRC) and deep-red colored hairy roots (DRC) were excised and transferred to the MS + 200 mg/L cefotaxim medium. The culture was kept at 25 °C under a 16 h light/8 h dark photoperiod. (Fig. 4C, D). To get more examples, we further chose two pale-red colored hairy roots PRC1 (Fig. 4C) and PRC2 (Additional file 2: Fig. S2A), three deep-red colored hairy roots DRC1 (Additional file 2: Fig. S2B), DRC2 (Additional file 2: Fig. S2C) and DRC3 (Fig. 4D), and a negative control hairy root P121 (Fig. 3B) were investigated the total content of anthocyanins using spectrophotometer (Fig. 5). Anthocyanins were detected in all hairy roots, but significantly higher in those transformed with *AmRosea1*. For example, anthocyanin contents were 0.773 mg/g.FW in PRC1 and 2.064 mg/g FW in DRC3 (Fig. 5). Moreover, anthocyanin content of 5 independent hairy roots were relatively higher than in a control hairy root P121, and the contents are being about 2.5- to 6.7-fold higher than control hairy root P121 (Fig. 5).

### Expression analysis of anthocyanin biosynthesis genes in hairy roots transformed with *AmRosea1*

**Fig. 6:**
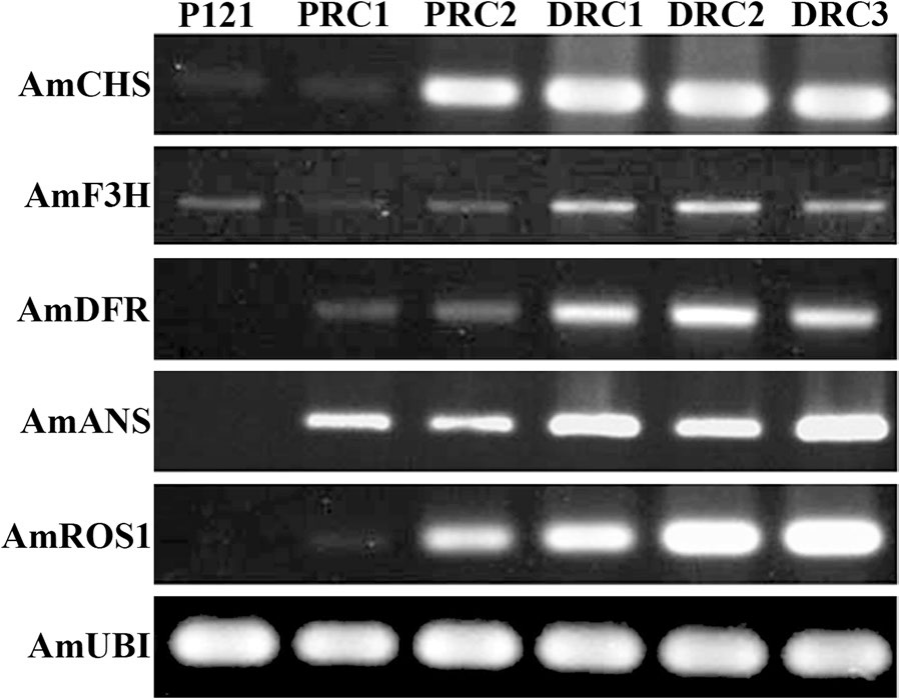
Comparison of the expression level of *AmCHS*, *AmF3H*, *AmDFR*, and *AmANS* between a negative control hairy root transformed with AR1193/pBI121 and five independent hairy roots transformed with AR1193/pBI35S:ROS1 by semi-quantitative RT-PCR analysis. P121, A negative control hairy roots transformed with AR1193/pBI121 (Fig. 2B); PRC1, 2 and DRC1-3, Five hairy roots transformed with AR1193 /pBI35S:ROS1 (Fig. 4C, D and Additional file 2: Fig. S2A, B, C)

We next investigated the relationship between AmRosea1, anthocyanin content and the expression levels of the main structural genes in the anthocyanin biosynthetic pathway. We chose two pale colored hairy root lines PRC1 and PRC2, three purple red colored hairy root lines DRC1, DRC2 and DRC3) and a negative control hairy root P121 to analyse the expression levels of *AmCHS*, *AmF3H*, *AmDFR*, and *AmANS* by semi-quantitative RT-PCR (Fig. 6). Clear expression of *AmCHS*, *AmF3H*, *AmDFR*, and *AmANS* were detected from five independent hairy roots. The expression levels of structural genes in DCR1, DCR2 and DCR3 lines were stronger than in PRC1 and PRC2 lines. Also, the expression patterns of *AmDFR* and *AmANS* coincided with the expression of *AmRosea1*. Moreover, the expression levels of *AmDFR* and *AmANS* seem to be harmony with anthocyanin accumulation in the hairy roots, (Fig. 6, Additional file 2: Fig. S2). Our findings suggest that *AmRos1* alone is able to stimulate structural gene expression in the anthocyanin biosynthetic pathway, and thereby induce anthocyanin accumulation in the hairy roots of *A.majus*.

## Discussion

The regulation of anthocyanin biosynthesis has been well examined in the above ground parts of plants such as flowers, leaves, seeds and fruits, in various species, but little has hitherto been known about underground organs such as roots, except in tuberous roots of sweet potato and potato (Liu et al. 2016; Strygina et al. 2019). In the present study, transformed hairy roots of *A. majus* proves to be an excellent model to investigate the regulatory mechanisms of anthocyanin biosynthesis and how anthocyanin synthesis can be regulated in the root by transcription factors *AmRosea1* and *AmDelila*. Previous studies have demonstrated that the bHLH gene, *AmDelila*, and the R2R3-MYB gene, *AmRosea1*, *AmRosea2* and *AmVenosa* are transcription factors and mainly involved in the control of anthocyanin biosynthesis in *A. majus* flowers (Goodrich et al. 1992; Schwinn et al. 2006; Shang et al. 2011). *Amdelila* results in loss of pigmentation only in the corolla tube, whereas *AmRosea1* promotes strong, intense red corolla pigmentation in the adaxial and abaxial epidermis of flowers (Goodrich et al. 1992; Schwinn et al. 2006). In the present study, we have detected that ectopic expression of *AmDelila* alone seems to be insufficient to affect the anthocyanin biosynthetic pathway, and therefore does not promote anthocyanin accumulation in transformed hairy roots of *A. majus* (Fig. 4A). In contrast, ectopic expression of *AmRosea1* alone clearly up-regulated expression of key structural target genes that are involved in the anthocyanin biosynthetic pathway (Fig. 6), thereby significantly promoting anthocyanin accumulation in transformed hairy roots (Fig. 4D, Additional file 2: Fig. S2), Similar results have been also detected in transformed hairy roots of both *Antirrhinum* and cotton when ectopic expression of *Rosea1* like R2R3-MYB gene *RLC1* (Gao et al. 2013). In this study, we also examined the levels of gene expression involved in the anthocyanin biosynthetic pathway and analysed anthocyanin accumulation from transformed roots of *A. majus* using *A. rhizogenes* AR1193/35S: ROS1. Although the anthocyanin content showed variations in the independent hairy roots, but still generate about 1.948, 1.959 and 2.064 mg/g FW high anthocyanin amounts were detected in the deep colour hairy roots (Fig. 5). This result suggests that hairy root induced by combination of *AmROS1* and *A. rhizogenes*-mediated transformation could prove to be an alternative approach for the production of anthocyanin compounds.

R2R3-MYB transcription factors are involved in regulation of tissue-specific anthocyanin accumulation in various plants, for example, *StAN1*, *StMYBA1*and *StMYb113* in potato (Liu et al. 2016); *LhMYB6* and *LhMYB12* in lily *(*Yamagishi et al. 2010); *Rosea1* and *Venosa* in *A. majus* (Schwinn et al. 2006; Shang et al. 2011); and AtMYB75 and AtMYB90 in *A. thaliana* (Borevitz et al. 2000). Also, two adjacent R2R3-MYB genes, *VvMYBA1* and *VvMYBA2* control the skin colour from red to white in grapes (Walker et al. 2007). In this study, we found that the structural genes *AmCHS, AmDFR* and *AmANS* were greatly upregulated in the transformed hairy roots of pBI35S:ROS1 (Fig. 6) and that these hairy roots showed notable anthocyanin accumulation (Fig. 4D, Additional file 2: Fig. S2). Moreover, the expression levels of an EBG gene *AmCHS*, and the LBG genes *AmDFR and AmANS* were harmony with expression of *AmRosea1*. Previous study had been demonstrated that the R2R3-MYB gene *AmRosea1* mostly involved in regulating LBGs *AmDFR* and *AmANS* expression in flower of *A. majus* (Schwinn et al. 2006). Therefore, the elevated expression of *AmCHS* might be the response of the metabolite feedback phenomenon induced by the up-regulation of LBGs. These results indicate that the genetic basis of root colour in *A. majus* is probably due to the silence of a common regulator of the *AmRosea1* or *AmRosea1*-like genes.

Several studies have reported that bHLH transcription factors like AmDelila constitute a group of regulatory genes involved in anthocyanin biosynthesis in plants (Goodrich et al. 1992; Xie et al. 2012). For instance, the insertion of a transposon in such a gene altered the flower tube color in *A. majus* (Goodrich et al. 1992) and altered flower color in the morning glory (Park et al. 2007). In *A. thaliana,* anthocyanin synthesis is controlled by three bHLHs AtTT8, AtGL3 and AtEGL3. The AtTT8 mutant has a low proanthocyanidins content in the pale-yellow seed coat and the expression of AtTT8 is highly correlated with the pigment content in the seed coat in *Arabidopsis* (Nesi et al. 2000). In apple, MdbHLH3 expression is in response to low temperature, and then, MdbHLH3 directly regulates the expression of MdMYB1. MdbHLH3 and MdMYB1 which then work together to activate anthocyanin biosynthesis (Xie et al. 2012). In the present study, we found that ectopic expression of *AmDelila* alone insufficient to affect the expression of the main endogenous anthocyanin synthesis genes, and therefore, the transformed hairy roots were showed no any coloration (Fig. 4A). These results suggest that expression of *AmDelila* alone does not stimulate the initiation of anthocyanin biosynthesis in roots. By contrary, expression of *AmRosea1* alone was able to activate *AmDFR* and *AmANS* expression and promoted anthocyanin synthesis, which was similar with co-expression of both AmRosea1 and AmDelila (Fig. 3; Fig. 6), indicating that *AmRosea1* may directly or indirectly affects expression of bHLH transcription factor *AmDelila* in the root. These results strongly suggest that *AmRosea1* or an *AmRosea1-like* R2R3-MYB gene plays a more important role than the bHLH gene *AmDelila* in regulating the initiation of anthocyanin biosynthesis in the root of *A. majus*.

In the present study, we investigated the action of transcription factors *AmRosea1* and *AmDelila* on anthocyanin synthesis in root of *A. majus*. Our results clearly show that *AmRosea1* alone is able to activate *AmDFR* and *AmANS* gene expression to enhance anthocyanin accumulation in the transformed hairy roots. these results excitingly suggest that *AmRosea1* is a useful tool to uniquely induce anthocyanins in the root of *A. majus*.

## Supplementary Information

**Additional file 1:Figure S1.** A simplified anthocyanin biosynthetic pathway in Antirrhinum. AmPAL, phenylalanine ammonia lyase; AmCHS, chalcone synthase; AmCHI, chalcone isomerase; AmF3H, flavanone-3-hydroxylase; AmF3′H, flavonoid-3′-hydroxylase; AmDFR, dihydroflavonol 4-reductase; AmANS, anthocyanidin synthase.

**Additional file 2:Figure S2.** Transformed hairy roots with AR1193/pBI35S:AmROS1. **A** Pale-red colored hairy root (PRC2); **B** Deep-red colored hairy root (DRC1); **C** Deep-red colored hairy root (DRC2). Scale bar = 1 cm.

## Data Availability

All data generated or analysed during this study are included in this published article and its Additional file 1, 2.

## Abbreviations

CHS: Chalcone synthase
CHI: Chalcone isomerase
F3H: Flavanone 3-hydroxylase
F3′H: Flavanoid 3′-hydroxylase
DFR: Dihydroflavonol-4-reductase
ANS: Anthocyanidin synthase
UFGT: Flavonoid3-O-glucosyl-transferase
PCR: Polymerase chain reaction
RT-PCR: Reverse transcription-polymerase chain reaction
NAA: 1-Naphthaleneacetic acid
CTAB: Hexadecyl trimethyl ammonium bromide

## References

[CR1] Ai TN, Naing AH, Arun M, Lim SH, Kim CK (2016). Sucrose-induced anthocyanin accumulation in vegetative tissue of *Petunia* plants requires anthocyanin regulatory transcription factors. Plant Sci.

[CR2] Albert NW, Davies KM, Lewis DH, Zhang H, Montefiori M, Brendolise C, Boase MR, Ngo H, Jameson PE, Schwinn KE (2014). A conserved network of transcriptional activators and repressors regulates anthocyanin pigmentation in eudicots. Plant Cell.

[CR3] Almeida J, Carpenter R, Robbins TP, Martin C, Coen ES (1989). Genetic interactions underlying flower colour patterns in *Antirrhinum majus*. Genes Dev.

[CR4] Borevitz JO, Xia YJ, Blount J, Dixon RA, Lamb C (2000). Activation tagging identifies a conserved MYB regulator of phenylpropanoid biosynthesis. Plant Cell.

[CR5] Cui M, Takayanagi K, Kamada H, Handa T (2001). Efficient shoot regeneration from hairy roots of *Antirrhinum majus* L. transformed by the *rol* type MAT vector system. Plant Cell Rep.

[CR6] Gao Z, Liu C, Zhang Y, Li Y, Yi K, Zhao X, Cui M (2013). The promoter structure differentiation of a MYB transcription factor *RLC1* causes red leaf coloration in empire red leaf cotton under light. PLoS ONE.

[CR7] Ghorbani A (2017). Mechanisms of antidiabetic effects of flavonoid rutin. Biomed Pharmacother.

[CR8] Gonzalez A, Zhao M, Leavitt JM, Lloyd AM (2008). Regulation of the anthocyanin biosynthetic pathway by the *TTG1/bHLH/Myb* transcriptional complex in *Arabidopsis* seedlings. Plant J.

[CR9] Goodrich J, Carpenter R, Coen ES (1992). A common gene regulates pigmentation pattern in diverse plant species. Cell.

[CR10] Jeziorek M, Sykłowska-Baranek K, Pietrosiuk A (2018). Hairy root cultures for the production of anti-cancer naphthoquinone compounds. Curr Med Chem.

[CR11] Kitamura S, Shikazono N, Tanaka A (2004). *TRANSPARENT TESTA**19* is involved in the accumulation of both anthocyanins and proanthocyanidins in *Arabidopsis*. Plant J.

[CR12] Kopustinskiene DM, Jakstas V, Savickas A, Bernatoniene L (2020). Flavonoids as anticancer agents. Nutrients.

[CR13] Liu Y, Lin-Wang K, Espley RV, Wang L, Yang H, Yu B, Dare A, Varkonyi-Gasic E, Wang J, Zhang J, Wang D, Allan AC (2016). Functional diversification of the potato R2R3 MYB anthocyanin activators *AN1*, *MYBA1*, and *MYB113* and their interaction with basic helix-loop-helix cofactors. J Exp Bot.

[CR14] Martin C, Carpenter R, Sommer H, Saedler H, Coen ES (1985). Molecular analysis of instability in flower pigmentation in *Antirrhinum majus*, following isolation of the pallida locus by transposon tagging. EMBO J.

[CR15] Martin C, Prescott A, Mackay S, Bartlett J, Vrijlandt E (1991). Control of anthocyanin biosynthesis in flowers of *Antirrhinum majus*. Plant J.

[CR16] Martin C, Gerats T (1993). Control of pigment biosynthesis genes during petal development. Plant Cell.

[CR17] Mehrotra S, Goel MK, Srivastava V, Rahman LU (2015). Hairy root biotechnology of *Rauwolfia serpentina*: a potent approach for the production of pharmaceutically important terpenoid indole alkaloids. Biotech Lett.

[CR18] Murashige T, Skoog F (1962). A revised medium for rapid growth and bioassays with tobacco tissue culture. Physiol Plantarum.

[CR19] Naing AH, Kim CK (2018). Roles of R2R3-MYB transcription factors in transcriptional regulation of anthocyanin biosynthesis in horticultural plants. Plant Mol Biol.

[CR20] Nesi N, Debeaujon I, Jond C, Pelletier G, Caboche M, Lepiniec L (2000). The *TT8* gene encodes a basic helix-loop-helix domain protein required for expression of *DFR* and *BAN* genes in *Arabidopsis siliques*. Plant Cell.

[CR21] Park KI, Ishikawa N, Morita Y, Choi JD, Hoshino A, Iida S (2007). A bHLH regulatory gene in the common morning glory, *Ipomoea purpurea*, controls anthocyanin biosynthesis in flowers, proanthocyanidin and phytomelanin pigmentation in seeds, and seed trichome formation. Plant J.

[CR22] Pelletier MK, Murrell JR, Shirley BW (1997). Characterization of flavonol synthase and leucoanthocyanidin dioxygenase genes in *Arabidopsis* (Further evidence for differential regulation of “early” and “late” genes). Plant Physiol.

[CR23] Rabino I, Mancinelli AL (1986). Light, temperature, and anthocyanin production. Plant Physiol.

[CR24] Ramsay NA, Glover BJ (2005). MYB–bHLH–WD40 protein complex and the evolution of cellular diversity. Trends Plant Sci.

[CR25] Ritala A, Dong L, Imseng N, Seppänen-Laakso T, Vasilev N, Krol S, Rischer H, Maaheimo H, Virkki A, Brändli J, Schillberg S, Eibl R, Bouwmeester H, Oksman-Caldentey KM (2014). Evaluation of tobacco (*Nicotiana tabacum* L. cv. Petit Havana SR1) hairy roots for the production of geraniol, the first committed step in terpenoid indole alkaloid pathway. J Biotech.

[CR26] Rogers SO, Bendich AJ (1985). Extraction of DNA from milligram amounts of fresh, herbarium and mummified plant tissues. Plant Mol Biol.

[CR27] Roy A (2021). Hairy root culture an alternative for bioactive compound production from medicinal plants. Curr Pharm Biotech.

[CR28] Saqallah FG, Hamed WM, Talib WH (2018). In vivo evaluation of *Antirrhinum majus* wound-healing activity. Sci Pharm.

[CR29] Schwinn K, Venail J, Shang YJ, Mackay S, Alm V, Butelli E, Oyama R, Bailey P, Davies K, Martin C (2006). A small family of MYB-regulatory genes controls floral pigmentation intensity and patterning in the genus *Antirrhinum*. Plant Cell.

[CR30] Senior I, Holford P, Cooley RN, Newbury HJ (1995). Transformation of *Antirrhinum majus* using *Agrobacterium rhizogenes*. J Exp Bot.

[CR31] Seo J, Lee J, Yang H, Ju J (2020). *Antirrhinum majus* L. flower extract inhibits cell growth and metastatic properties in human colon and lung cancer cell lines. Food Sci Nutri.

[CR32] Shang Y, Venail J, Mackay S, Bailey PC, Schwinn KE, Jameson PE, Martin CR, Davies KM (2011). The molecular basis for venation patterning of pigmentation and its effect on pollinator attraction in flowers of *Antirrhinum*. New Phytol.

[CR33] Sharma A, Verma P, Mathur A, Mathur AK (2018). Genetic engineering approach using early Vinca alkaloid biosynthesis genes led to increased tryptamine and terpenoid indole alkaloids biosynthesis in differentiating cultures of *Catharanthus roseus*. Protoplasma.

[CR34] Shen WJ, Forde BG (1989). Efficient transformation of Agrobacterium spp. by high voltage electroporation. Nucleic Acids Res.

[CR35] Sommer H, Saedler H (1986). Structure of the chalcone synthase gene of *Antirrhinum majus*. Mol Gent Genomics.

[CR36] Strygina KV, Kochetov AV, Khlestkina EK (2019). Genetic control of anthocyanin pigmentation of potato tissues. BMC Genet.

[CR37] Tavsan Z, Kayali HA (2019). Flavonoids showed anticancer effects on the ovarian cancer cells. Biomed Pharm.

[CR38] Thakore D, Srivastava AK (2017). Production of biopesticide azadirachtin using plant cell and hairy root cultures. Eng Life Sci.

[CR39] Walker AR, Lee E, Bogs J, Mc David DAJ, Thomas MR, Robison SP (2007). White grapes arose through the mutation of two similar and adjacent regulatory genes. Plant J.

[CR40] Winkel-Shirley B (2001). Flavonoid biosynthesis. A colorful model for genetics, biochemistry, cell biology, and biotechnology. Plant Physiol.

[CR41] Xie XB, Li S, Zhang RF, Zhao J, Chen YC, Zhao Q, Yao YX, You CX, Zhang XS, Hao YJ (2012). The bHLH transcription factor *MdbHLH3* promotes anthocyanin accumulation and fruit colouration in response to low temperature in apples. Plant Cell Environ.

[CR42] Yamagishi M, Shimoyamada Y, Nakatsuka T, Masuda K (2010). Two R2R3-MYB genes, homologs of petunia *AN2*, regulate anthocyanin biosynthesis in flower tepals, tepal spots and leaves of Asiatic hybrid lily. Plant Cell Physiol.

[CR43] Yousefian S, Lohrasebi T, Farhadpour M, Haghbeen K (2020). production of phenolic acids in hairy root cultures of medicinal plant *Mentha spicata* L. in response to elicitors. Mol Biol Res Commun.

[CR44] Zhang Y, Butelli E, Martin C (2014). Engineering anthocyanin biosynthesis in plant. Curr Opin Plant Biol.

