# Supplementary figures and images for: The combination of R2R3-MYB gene AmRosea1 and hairy root culture is a useful tool for rapidly induction and production of anthocyanins in Antirrhinum majus L
Source: AMB Express. 2021 Sep 14;11:128. doi: 10.1186/s13568-021-01286-6 (PMC8440734; doi:10.1186/s13568-021-01286-6)

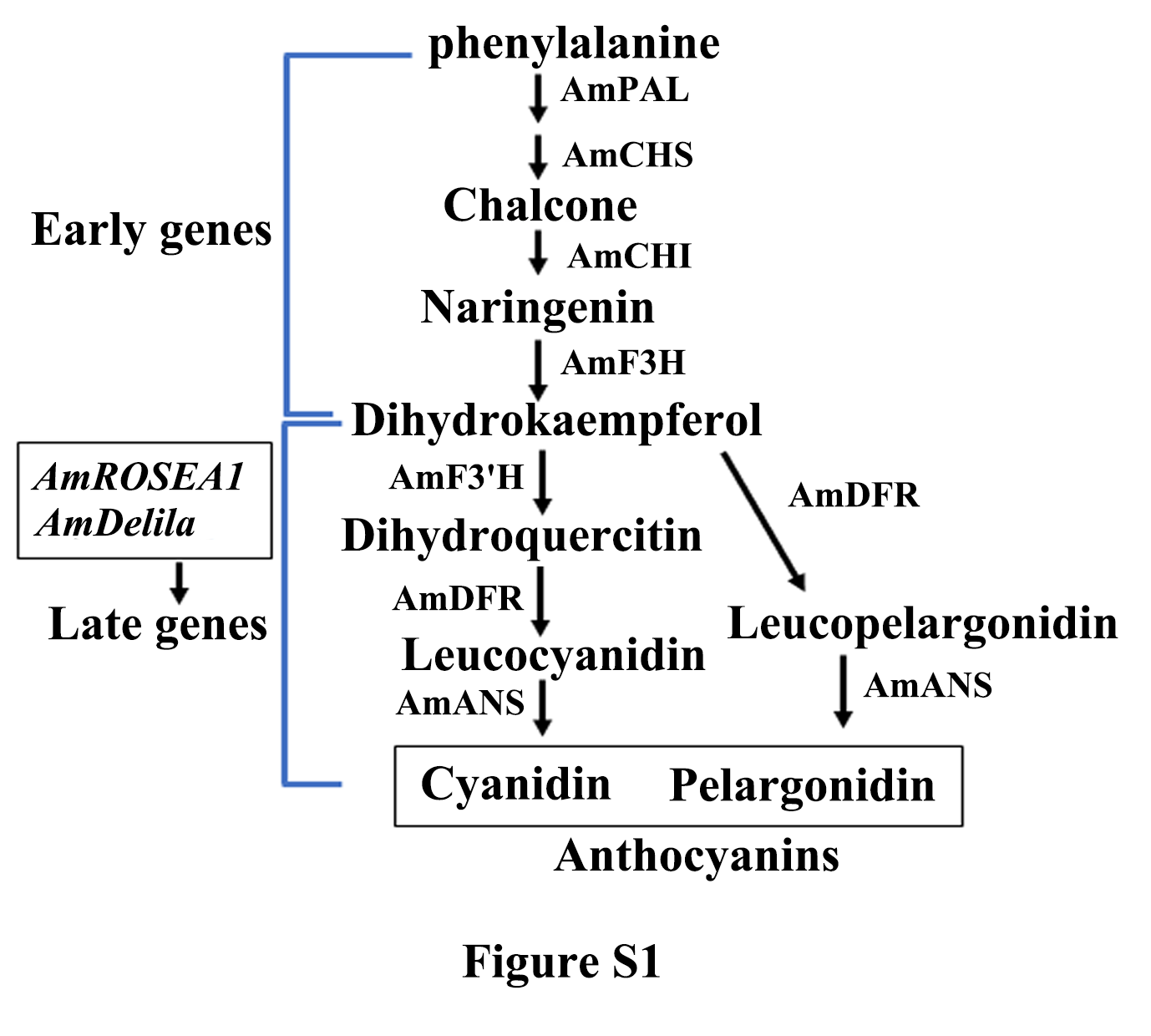

Supplement: Supplementary file 1 — Additional file 1:Figure S1. A simplified anthocyanin biosynthetic pathway in Antirrhinum. AmPAL, phenylalanine ammonia lyase; AmCHS, chalcone synthase; AmCHI, chalcone isomerase; AmF3H, flavanone-3-hydroxylase; AmF3′H, flavonoid-3′-hydroxylase; AmDFR, dihydroflavonol 4-reductase; AmANS, anthocyanidin synthase. [file 13568_2021_1286_MOESM1_ESM.tif]

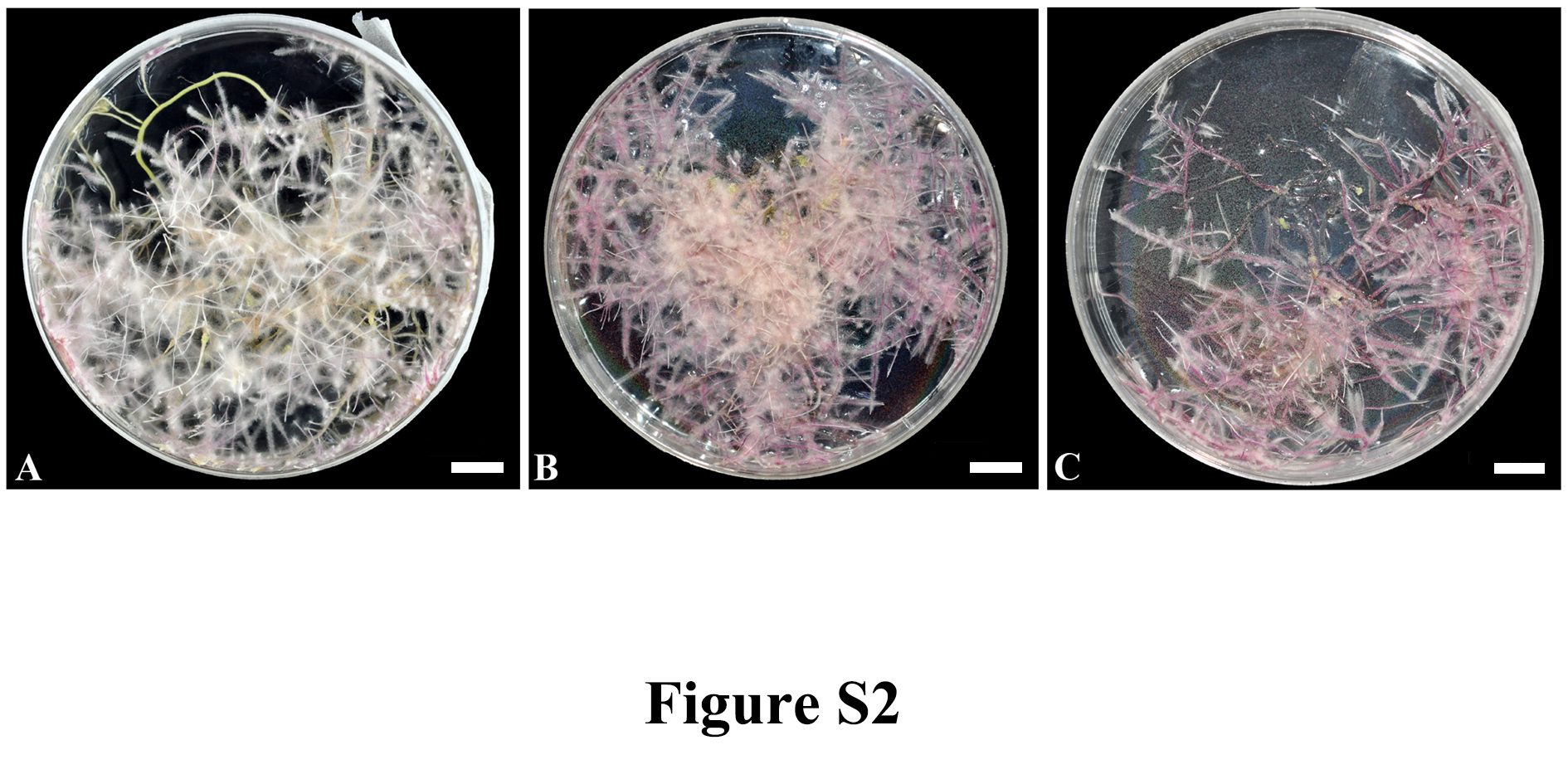

Supplement: Supplementary file 2 — Additional file 2:Figure S2. Transformed hairy roots with AR1193/pBI35S:AmROS1. A Pale-red colored hairy root (PRC2); B Deep-red colored hairy root (DRC1); C Deep-red colored hairy root (DRC2). Scale bar = 1 cm. [file 13568_2021_1286_MOESM2_ESM.jpg]
